# The Interaction between Laminin-332 and α3β1 Integrin Determines Differentiation and Maintenance of CAFs, and Supports Invasion of Pancreatic Duct Adenocarcinoma Cells

**DOI:** 10.3390/cancers11010014

**Published:** 2018-12-21

**Authors:** Ana C. Martins Cavaco, Maryam Rezaei, Michele F. Caliandro, Augusto Martins Lima, Martin Stehling, Sameer A. Dhayat, Jörg Haier, Cord Brakebusch, Johannes A. Eble

**Affiliations:** 1 Institute of Physiological Chemistry and Pathobiochemistry, University of Münster, Waldeyerstr. 15, 48149 Münster, Germany; acmcavaco@gmail.com (A.C.M.C.); mrezaei@uni-muenster.de (M.R.); michele.caliandro@stud.unifi.it (M.F.C.); augustomlima@gmail.com (A.M.L.); 2 Department of Cell and Developmental Biology, Max Planck Institute for Molecular Biomedicine, Röntgenstraße 20, 48149 Münster, Germany; martin.stehling@mpi-muenster.mpg.de; 3 Department of General, Visceral and Transplantation Surgery, University Hospital Münster, Albert-Schweitzer-Campus 1 (W1), 48149 Münster, Germany; sameer.dhayat@ukmuenster.de (S.A.D.); joerg.haier@nordakademie.de (J.H.); 4 Biotech Research and Innovation Center (BRIC), University of Copenhagen, Ole Maaløes vej 5, 2200 Copenhagen, Denmark; cordbrakebusch@gmail.com

**Keywords:** pancreatic cancer, cancer-associated fibroblasts (CAF), differentiation, laminin-332, integrin α3β1, spheroid culture, invasion

## Abstract

Ranking among the most lethal tumour entities, pancreatic duct adenocarcinoma cells invade neighbouring tissue resulting in high incidence of metastasis. They are supported by tumour stroma fibroblasts which have undergone differentiation into cancer-associated fibroblasts (CAFs). Stiffness of cell substratum, cytokines, such as transforming growth factor-β (TGF-β), and stromal matrix proteins, such as laminin-332, are factors which promote CAF differentiation. In a spheroid culture system, differentiation of CAFs was analysed for laminin-332 production, laminin-binding integrin repertoire, adhesion and migration behaviour, and, in heterospheroids, for their interplay with the pancreatic duct adenocarcinoma AsPC-I cells. Our data reveal that CAFs produce laminin-332 thus contributing to its ectopic deposition within the tumour stroma. Moreover, CAF differentiation correlates with an increased expression of α3β1 integrin, the principal laminin-332-receptor. Beyond its role as novel CAF marker protein, integrin α3β1 crucially determines differentiation and maintenance of the CAF phenotype, as knock-out of the integrin α3 subunit reversed the CAF differentiated state. AsPC-I cells co-cultured in heterospheroids with integrin α3-deficient CAFs invaded less than from heterospheroids with wild-type CAFs. This study highlights the role of integrin α3β1 integrin-laminin-332 interaction of CAFs which promotes and sustains differentiation of CAFs and promotes carcinoma invasion.

## 1. Introduction

Pancreatic duct carcinoma is one of the most lethal diseases among malignant cancers. It develops from epithelial cells, which form a monolayer lining the ducts of the exocrine pancreas and are anchored to the underlying basement membrane [1]. The basement membrane is a specialized sheet-like structure of the extracellular matrix (ECM), which separates epithelial, endothelial, neural, muscle, and fat cells from the fibroblasts embedded in the connective tissue [2]. Laminins, typical components of the basement membrane [3], consist of three chains, α, β, and γ that trimerize into a molecule of three short arms, a long arm shaped by the coiled-coil trimerization domain and the globular G-domain. The first three of five subdomains, LG1-LG3, of the G-domain harbour the interaction site of most cellular laminin-receptors and it is blocked by the monoclonal antibody BM2 [4]. Laminin-332 stands apart from other members of the laminin family structurally and functionally [5]. It is located as anchoring filaments within the lamina lucida of basement membranes underneath the epidermis and epithelial cells lining a gland duct [4]. Produced by epithelial and carcinoma cells, laminin-332 anchors cells to the underlying basement membrane and allows migration [6,7]. Proteolytic cleavage within the G-domain releases the heparansulfate-binding C-terminal LG4-LG5 tandem domain [8,9]

Cellular contacts with laminin-332 are mediated by laminin-binding members of the integrin family [10]. Integrins are heterodimeric transmembrane proteins, consisting of two subunits, α and β. They bind to ECM ligands with their extracellular domains and are connected to the cytoskeleton via their cytoplasmic domains. Thus, the β1 integrin transmit mechanical forces from the actomyosin system to the ECM. The integrins α6β1 and α6β4 bind laminin-332 [7,11]. In comparison, integrin α3β1 has higher selectivity for this ECM protein [9,12]. The integrin α3β1 and the hemidesmosome integrin α6β4 seem to play a role in tumorigenesis and progression of carcinoma, albeit along different pathways [13,14,15]. The disintegrin lebein-1, isolated from the venom of the snake *Vipera lebetina*, was identified to prevent laminin-binding to their respective integrin receptors [16]. 

Just as important as the cancer cells in solid tumours is the microenvironment, consisting of fibroblastic stromal cells, endothelial cells from tumour-induced angiogenesis, and immune cells. Such stromal fibroblasts are not only bystanders but differentiate into cancer-associated fibroblasts (CAFs) under the influence of neighbouring tumour cells [17,18,19]. Then, they support tumour cell progression and invasion [20]. These CAFs have a similar morphology to myofibroblast during wound healing or fibrotic diseases [21]. However, a definite set of immunologic markers to identify CAFs is not entirely established, maybe due to the heterogeneity of CAF subpopulations [17]. General marker for CAFs are the intracellular α-smooth muscle actin (αSMA), as well as the membrane-anchored platelet-derived growth factor (PDGF) receptor β [18] and neural/glial antigen 2 (NG2) proteoglycan (chondroitin-4-sulfate proteoglycane-4, CSPG-4) [22]. Albeit the mechanism is not entirely understood, different parameters, such as matrix stiffness, paracrine and autocrine growth factors, may trigger differentiation of fibroblasts into CAFs [23,24,25]. Among the growth factors, transforming growth factor-β (TGF-β) is a major inducer of CAF differentiation and can be released from its storage sites within ECM proteins in a tension-dependent manner [18,25,26]. CAFs synthesize most ECM components, some of which are typical of the tumour stroma, such as tenascin-W and laminin-332 chains [27,28]. In this respect, laminin-332 shows two worthwhile features. It is ectopically expressed in different tumour tissue, among them pancreatic duct adenocarcinoma [6,29]. Moreover, single chains of laminin-332, especially the laminin β3 and γ2, are deposited within the tumour stroma or are even detectable in the blood of tumour patient with poor prognosis [7,30,31].

Here, we unravel a role of laminin-332 and its cellular interaction via α3β1 integrin in CAF differentiation. Our experiments demonstrate that this interaction has a crucial role in inducing CAF differentiation and in sustaining their phenotypic status. Furthermore, integrin α3β1 on CAFs and the ectopically deposited laminin-332 are not only markers of CAF differentiation but also determine the fate of CAFs and, due to their supportive role of cancer invasion, also promote carcinoma invasion.

## 2. Results

### 2.1. Pancreatic Tumour Stroma Is Characterized by Ectopic Expression of Laminin-332 and by the Presence of Cancer-Associated Fibroblasts (CAFs)

**Figure 1:**
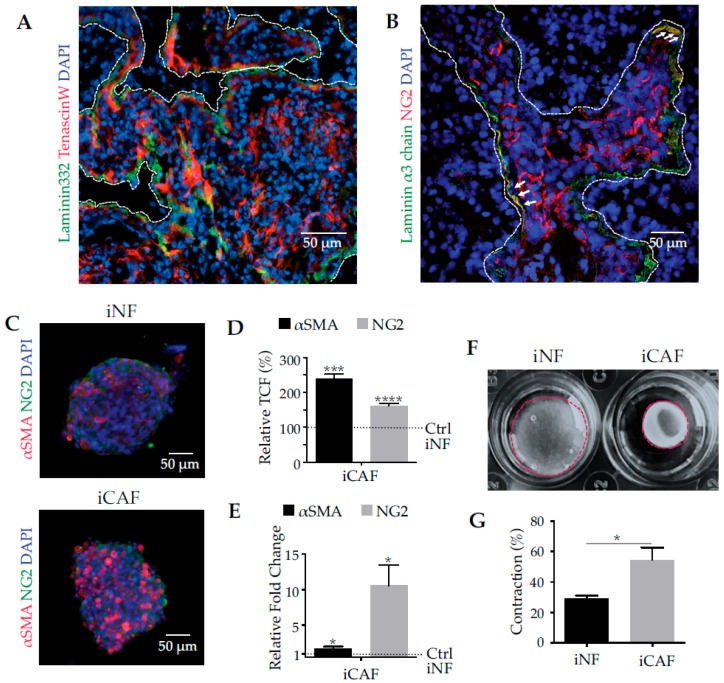
Pancreatic tumour tissue in situ and spheroid cultures of normal and cancer-associated fibroblasts as in an in vitro model. In (**A**,**B**) histological sections of human biopsies of pancreatic adenocarcinoma grade III, were stained for laminin-332, tenascin-W and the CAF-marker neural/glial antigen 2 (NG2) and pictures were taken with laser scanning microscopy. (**A**) Laminin-332 co-localizes with tenascin-W, a tumour stroma marker, and is prevalently deposited at the invasion front of the tumour. The tumour stroma is delineated by the white dashed line. (**B**) NG2, a cell-bound marker for cancer-associayed fibroblasts (CAFs), was found near laminin-332-deposits, denoted by the white arrows. (**C**) Spheroids of immortalized normal fibroblasts (iNFs) and immortalized cancer-associated fibroblasts (iCAFs), which had been isolated from healthy pancreatic tissue and from human pancreatic adenocarcinoma biopsies, respectively, and immortalised, were cultivated for 24 h and immunohistochemically analysed for CAF marker proteins, NG2 and α-smooth muscle actin (αSMA), of CAFs. Quantification of CAF markers, αSMA and NG2, in homospheroids of pancreatic iCAFs and the normal counterpart, iNFs at both protein level detected by immunofluorescence (**D**) and at transcriptional level analysed by qPCR (**E**). The IF staining quantification in (**D**) shows the relative percentage of total corrected fluorescence as compared to the control, iNFs, which was considered 100%. The quantification of the transcriptional levels in (**E**) is shown as relative fold of change as compared to the control, iNFs, which was considered 1. (**F**) iCAFs contracted collagen-I gels significantly stronger than iNFs. (**G**) Contraction was calculated as the gel shrinkage normalised to the original gel size. Means ± SEM of three independent experiments are shown. Biometric data in (**D**,**E**,**G**) were statistically evaluated by *t*-test (*, *p* < 0.05; ***, *p* < 0.001; ****, *p* < 0.0001).

To examine the composition of the tumour stroma of human pancreatic adenocarcinoma, we stained histological sections of pancreatic cancer patients for laminin-α3 chain and for tenascin-W, both markers for the extracellular matrix (ECM) (Figure 1A), as well as for NG2, a characteristic marker of CAFs (Figure 1B). Both extracellular proteins are present in the tumour stroma, albeit with a distinct distribution. Tenascin-W was deposited throughout the tumour bulk (Figure 1A) and, despite some colocalization, laminin-332 was prevalently deposited at the border of the tumour stroma, where tumour cell invasion occurs (Figure 1A). Moreover, CAFs were in the vicinity of these deposited matrix proteins, suggesting that laminin-332 is involved in the interplay between CAFs and cancer cells (Figure 1B). To obtain a more detailed characterization of these cells, CAFs from a surgical pancreatic cancer specimen were isolated and immortalized by lentiviral transfection with human telomerase (hTERT). The immortalized CAFs (iCAFs) were compared to immortalized human pancreatic fibroblasts (iNFs), the normal counterparts from a healthy donor.

Cultivation of stromal fibroblasts under common cell culture conditions caused several problems. The stiff plasticware, on which adherent cells were usually cultured, stimulated fibroblasts to express αSMA, a typical marker of activated fibroblasts and CAFs [23,25]. When grown on hydrogels of different stiffness, fibroblasts differentiated into CAFs in a matrix stiffness-dependent manner [25]. To study CAF differentiation independently of matrix stiffness, iNFs and iCAFs, were cultivated as spheroids and analysed for CAF markers, αSMA and NG2, by immunofluorescence staining (Figure 1C). Although iNFs express both marker proteins, the expression of these proteins is significantly increased in iCAFs (Figure 1D). The immunofluorimetric quantification of protein expression was corroborated at the transcriptional level, with qPCR. As compared to the iNFs, iCAFs have upregulated mRNA levels of αSMA and NG2 by almost 2-fold and even 10-fold, respectively (Figure 1E). Functionally, CAFs are characterized by their increased capability to exert mechanical forces onto their surrounding ECM. Embedded into a gel of collagen-I, iCAFs contracted the gel dramatically stronger than the iNFs (Figure 1F,G), thereby proving that the iCAFs not only showed characteristic CAF markers but also functionally exerted more mechanical forces than iNFs.

### 2.2. Comparison of Normal Fibroblasts and CAFs from Pancreatic Tumour Stroma Reveals That Integrin α3β1 and Laminin-332 Are Differentiation Markers 

**Figure 2:**
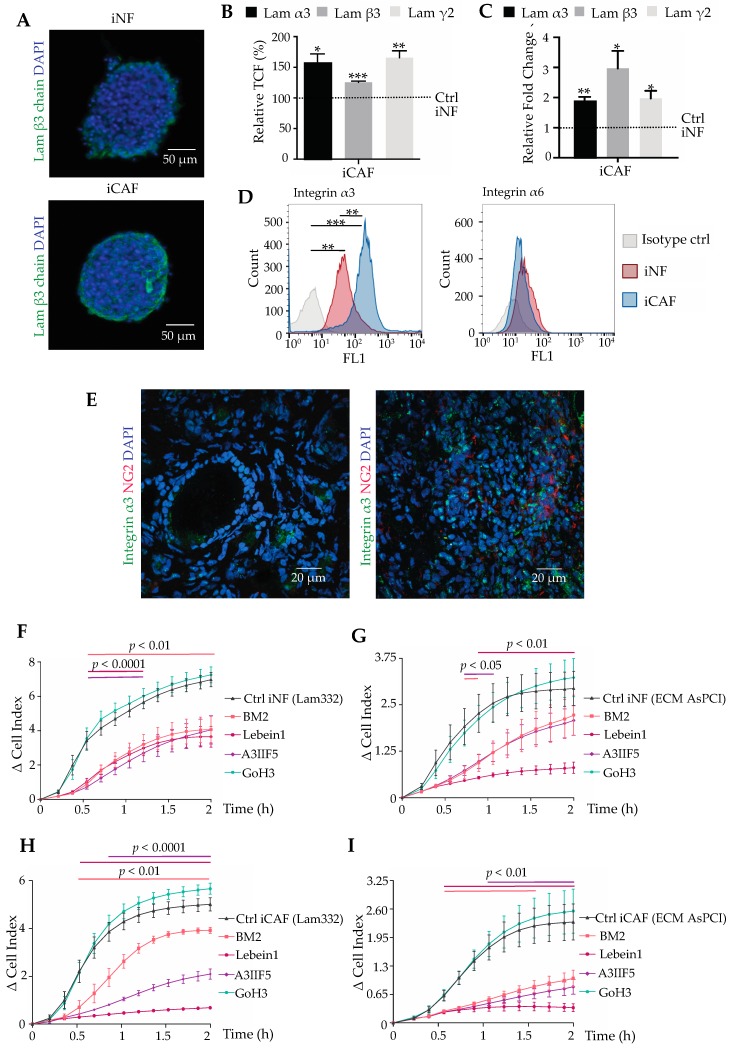
iCAFs express more laminin-332 and integrin α3β1 than iNFs in spheroid culture. (**A**) Spheroids of iCAFs and of iNFs, cultivated for 24 h, were stained with antibodies against the three chains of laminin-332 (representative images of the β3 chain are shown). All three chains of laminin-332 were produced by both iNFs and iCAFs, but expression was significantly upregulated in iCAFs at both protein (**B**) and transcriptional levels (**C**). Protein expression was quantified as total corrected fluorescence from immunofluorescence images and normalized to the control values in iNF spheroids, which were considered 100% (*, *p* < 0.05; **, *p* < 0.01; ***, *p* < 0.001). The transcriptional levels in (**C**) were quantified by qPCR and the relative fold of change was compared to the control, iNFs, which was considered 1. (**D**) Flow cytometric quantification of integrin subunits, α3 and α6, subunits of the laminin-binding integrins, α3β1, α6β1, and α6β4. Integrin α3β1, but not the α6 subunit-containing integrins are upregulated in iCAFs as compared to iNFs. Significance was determined by comparing mean fluorescence intensities (**, *p* < 0.01; ***, *p* < 0.001). (**E**) Normal and carcinoma-affected pancreas tissue in the left and right panels, respectively, were stained by immunofluorescence for integrin α3 subunit (green) along with the CAF marker NG2 (red). The intenser staining of both proteins in the right panel indicates an upregulation of integrin α3β1 in the pancreatic carcinoma tissue and its CAFS. (**F**,**G**) Adhesion of iNFs on laminin-332 coated surface and AsPC-I-deposited ECM, respectively, and (**H**,**I**) adhesion of iCAFs on laminin-332-coated surface and on AsPC-I-deposited ECM, respectively, were recorded by real time impedance measurements. Adhesion experiments were carried out in the absence and presence of different inhibitors blocking laminin-332 (BM2, 20 μg/mL), laminin-binding integrins (lebein1, 10 μg/mL), integrin α3β1 (A3IIF5, 10 μg/mL) or integrin α6 subunit-containing integrins (GoH3, 10 μg/mL). Means ± SEM of three independent experiments are shown, and adhesion values at every time point were compared by *t*-test. The time windows in which the adhesion signal differed significantly from the non-inhibited control is shown as lines in the same colour above the plots with the significance.

Histological sections of pancreatic adenocarcinoma tissue revealed the presence of ectopically expressed laminin-332 in the tumour stroma. To identify, whether normal fibroblasts or CAFs are potential sources of laminin-332, spheroids of iNFs and iCAFs were also analysed for expression of the three laminin-332 chains, α3, β3, and γ2, by immunofluorescence (Figure 2A) and by qPCR. At both protein and transcriptional level, iCAFs synthesized significantly more laminin-332 chains as compared to their normal counterparts (Figure 2B,C). Among the laminin-binding integrins with affinity to laminin-332, integrin α3 subunit is expressed on the surface of iNFs and iCAFs at high levels. Additionally, the integrin α6 subunit was detected on the cells (Figure 2D). Moreover, integrin α3 is significantly up-regulated during the differentiation process with remarkably higher expression in iCAF than in iNFs. In contrast, integrin α6 expression remained almost unchanged between iCAFs and iNFs. These results suggested, that integrin α3β1 is a marker for CAF differentiation along with the expression and deposition of its ligand, laminin-332. In situ, integrin α3 subunit is also upregulated along with the CAF marker NG2 in pancreatic cancer tissue as compared to normal pancreas tissue (Figure 2E).

The adhesion of both iNFs and iCAFs to laminin-332 or laminin-332-containing ECM, which had been deposited by AsPC-I cells, was examined in a real-time impedance-based assay, using the xCELLigence system. When seeded onto laminin-332, iNFs and iCAFs adhered and spread intensely on the laminin-332 within two hours (Figure 2F,H). So did they on AsPC-I-deposited ECM (Figure 2G,I). The integrin α6 subunit -blocking antibody GoH3 failed to block adhesion of both cell types on both substrates. In contrast, the integrin α3 subunit-blocking antibody A3IIF5 significantly inhibited adhesion of iNFs, but even more of iCAFs, underlining that α3β1 integrin was predominantly responsible for the interaction with laminin-332. This is in line with the upregulated integrin α3 subunit expression in iCAFs. The integrin α3-blocking antibody blocked adhesion of iNFs and iCAFs on laminin-332 at least as strong as the laminin-332-blocking antibody BM2, indicating that the α3β1 integrin-laminin-332 interaction is mainly responsible of the cellular adhesion in this assay. Along the same line, lebein-1, a snake venom-derived disintegrin that effectively inhibits laminin-binding integrins, showed a persistent inhibition of iCAFs adhesion on both laminin-332 and AsPC-I-deposited matrix.

### 2.3. Stimulation of Pancreatic CAF Differentiation by TGF-β1 Depends on Laminin-332-Integrin Binding

**Figure 3:**
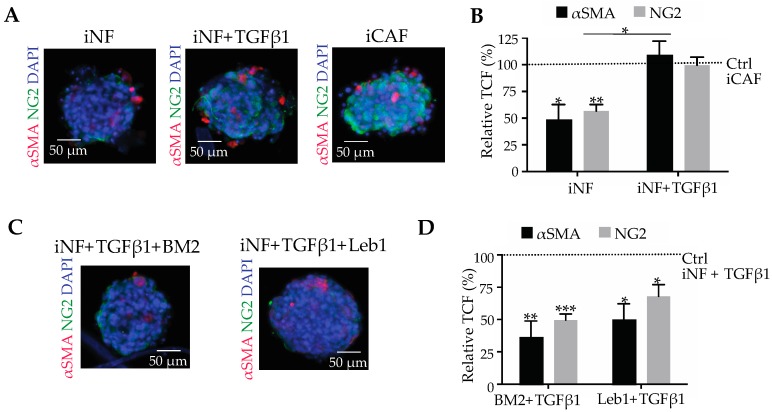
TGF-β1 induces iNFs to express CAF markers, αSMA and NG2, a process, in which the laminin-332-integrin interaction plays a role. (**A**) After stimulation with TGF-β1 (10 ng/mL) for 12 h, spheroids of iNFs upregulated the expression of αSMA and NG2 to a similar extent as iCAF spheroids, as demonstrated by the immunofluorescent staining. (**B**) Immunofluorescent staining in (**A**) was quantified and normalized to the staining intensity of iCAF spheroids to yield the relative total corrected fluorescence, which was considered 100%. (**C**) When BM2 (20 μg/mL) or lebein1 (10 μg/mL), which distinctly interfere with laminin-332-integrin interactions, were added to iNFs and simultaneously stimulated with TGF-β1 (10 ng/mL), the expression of αSMA and NG2 significantly decreased. (**D**) Quantification of immunofluorescence staining and normalization to non-inhibited TGF-β1-stimulated iNF spheroids suggested that blockage of the integrin-mediated cell interaction with laminin-332 reduces TGF-β1-induced CAF differentiation. In (**B**,**D**), mean values ± SEM of three independent experiments are shown and compared with *t*-test (*, *p* < 0.05; **, *p* < 0.01; ***, *p* < 0.001).

In spheroid culture, iNFs retained their phenotype with low levels of the differentiation markers, αSMA and NG2, which are upregulated up stimulation with TGF-β1, a cytokine that triggers CAF differentiation (Figure 3A). The corrected total fluorescence values reached by the TGF-β1-treated iNF were comparable to the ones of iCAFs, which were also cultured in spheroids but in the absence of exogenous TGF-β1 (Figure 3A,B).

To study the role of laminin-332 and its integrin receptors, in TGF-β1-dependent CAF differentiation, iNFs from normal pancreatic tissue were cultivated as spheroids and treated with TGF-β1 in the absence or presence of inhibitors of the integrin-mediated cell interactions with laminin-332. If TGF-β1-stimulated iNF spheroids were treated with the monoclonal antibody BM2 which blocks accessibility of laminin-332 to any potential integrin binding partner, the expression of both CAF-markers significantly decreased and approached almost the levels of non-TGF-β-treated control (Figure 3C,D). Complementarily, treatment of TGF-β1-stimulated iNFs with lebein-1, that effectively inhibits laminin-binding integrins, also reduced the expression of αSMA and NG2 to approximately the same extent as BM2 (Figure 3C,D). These data suggested that blockage of the integrin-laminin-332 interaction interaction is important in TGF-β1-stimulated CAF differentiation.

### 2.4. Laminin-332-Integrin Interaction Sustains the CAF Phenotype 

**Figure 4:**
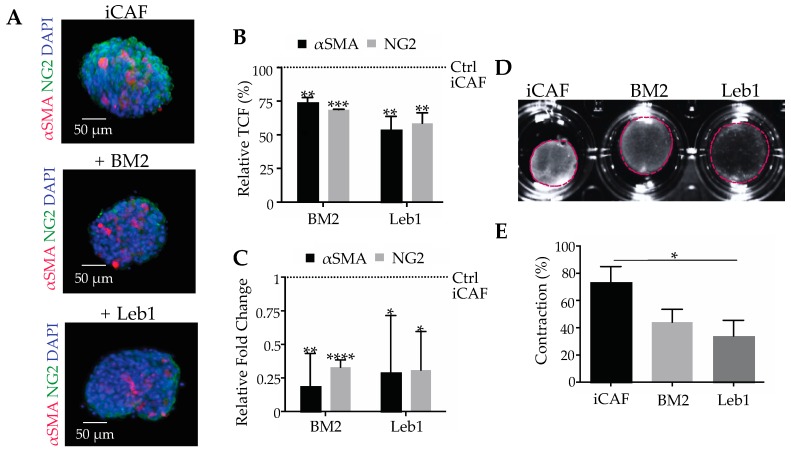
Laminin-332-integrin interaction is essential not only for differentiation, but also maintaining the differentiated phenotype of iCAFs. (**A**) Spheroids of iCAFs were treated with BM2 (20 μg/mL) or lebein-1 (10 μg/mL) for 12 h to block the integrin binding site in laminin-332 or the laminin-binding integrins, respectively, before they were immunofluorimetrically stained for αSMA and NG2. (**B**) Quantification of the immunofluorescent signals and normalization to the corresponding signals of non-treated iCAF spheroids revealed a significant reduction of both CAF markers after inhibition of the laminin-332-integrin interaction, iCAFs signals were considered 100%. (**C**) The expression of the two CAF markers at the transcriptional level was quantified by qPCR and normalized to the non-treated spheroids of iCAFs, which expression was considered 1. (**D**) iCAFs embedded in collagen-I gels dramatically reduced contraction in the presence of BM2 (20 μg/mL) or lebein-1 (10 μg/mL). (**E**) Gel contraction was quantified as reduction of gel area normalized to the initial gel size. Blockage of laminin-332-integrin interaction compromised force exertion and gel contraction of iCAFs. In (**B**,**C**,**E**), means ± SEM of three independent experiments are shown and compared with a *t*-test for statistical analysis purposes (*, *p* < 0.05; **, *p* < 0.01; ***, *p* < 0.001; ****, *p* < 0.0001).

To assess the effect of blocking the interaction of laminin-332 and its cognate integrin receptors on already differentiated CAFs, spheroids of iCAFs were treated with BM2 or lebein-1 (Figure 4). Due to its abundance on iCAFs, integrin α3β1 likely was the main target of lebein-1 among the laminin-binding integrins. The inhibitors were applied either individually or in combination. Whereas untreated iCAFs express αSMA and NG2 abundantly, the expression of both markers decreased significantly when treated with either BM2 or lebein-1 (Figure 4A) at the immunofluorimetrically detected protein level (Figure 4B) and at the transcriptional level (Figure 4C). To test whether the altered gene expression is accompanied with differences in cell function, the ability of iCAFs to contract a collagen gel was tested in the presence of BM2 and lebein1 (Figure 4D). Whereas the untreated iCAFs contracted the collagen gel by approximately 73% of its initial size, inhibition of the integrin-laminin-332 interaction with BM2 and lebein-1 significantly diminished gel contraction, with contraction percentages of approximately 44% and 34%, respectively (Figure 4E). This inhibitory effect on force transmission onto the collagen matrix depends on the iCAF differentiation state. Moreover, the combination of BM2 and lebein-1 did not show a hyperadditive effect (Figure S1). This lack of synergism ruled out interactions other than the mutual binding between laminin-332 and its integrin receptors which might be responsible for the differentiation state of CAFs. The fact that already differentiated iCAFs, upon treatment with BM2 or lebein-1, reversed their phenotype to the one of normal fibroblasts, strongly suggested that the laminin-332-integrin α3β1 interaction is also essential in sustaining the already differentiated CAF phenotype.

### 2.5. Interacting with Laminin-332, Integrin α3β1 Is Not Only a Marker, but also a Player on CAF Differentiation

**Figure 5:**
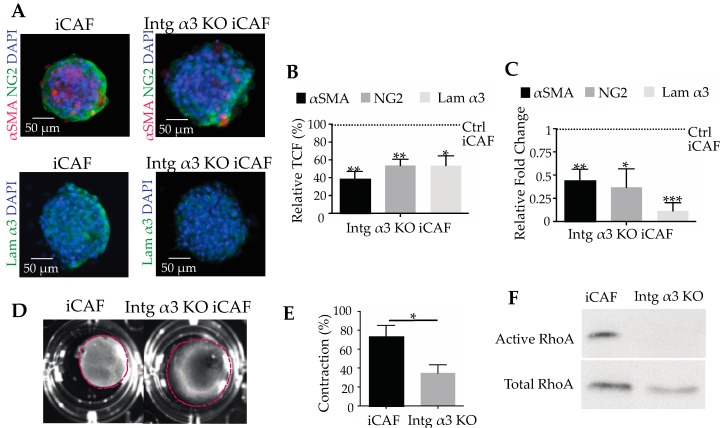
Integrin α3β1 is the laminin-332 receptor, which is essential for pancreatic CAFs differentiation. (**A**) Spheroids of cancer-associated fibroblasts either expressing or lacking integrin α3β1, iCAFs and α3 KO-iCAFs were immunofluorimetrically compared for their expression of the cellular CAF markers, αSMA and NG2, or for their expression of laminin-332. (**B**) The immunofluorimetric signals of the α3KO-iCAF spheroids were normalized to the ones in integrin α3β1 integrin-expressing iCAFs, which were considered 100%. (**C**) At the transcriptional level, the expression of CAF markers and of laminin α3-chain was quantified and normalized to the values of the iCAFs spheroids, which expression was considered 1. At both protein and transcriptional level, the reduction of CAF markers and of laminin-332 shows that CAF differentiation essentially depends on the cellular interaction via α3β1 integrin with its ligand laminin-332. (**D**) Collagen contraction assay, and (**E**) its biometric quantification demonstrated that integrin α3KO-iCAFs contracted the collagen-I gel significantly less than iCAFs which bear the integrin α3β1, although this integrin does not bind to collagen-I. (**F**) GTP-bound RhoA of iCAFs and of integrin α3β1-deficient iCAFs (α3KO-iCAFs) was determined by pull-down and compared to the total cellular amount of RhoA. In line with their low contractibility, α3KO-iCAFs show a low activity of RhoA, indicating that the integrin α3β1-laminin-332 interaction of CAFs indirectly activates the actomyosin system of iCAFs and increases cellular force exertion, which is transmitted via other integrins to the collagen-I gel. Means ± SEM of three independent experiments are shown and compared with *t*-test (*, *p* < 0.05; **, *p* < 0.01; ***, *p* < 0.001). The RhoA pull-down assay in (**F**) was performed in 2 independent experiments.

To prove the key role of integrin α3β1 and its interaction with laminin-332 in CAF differentiation, α3KO-iCAFs were generated by CRISPR/Cas9 technology. Flow cytometry of these cells revealed that knocking-out integrin α3 did not change the integrin subunit abundance of β1, but slightly increased the expression of α6 subunit (Figure S2). The expression of the collagen-binding integrin α2β1, presumably involved in collagen gel contraction, did not change significantly after knocking out the integrin α3 gene (Figure S2). Importantly, when grown in spheroids, the α3KO-iCAFs clearly differed from the parental iCAFs in the expression of differentiation marker proteins (Figure 5A). All tested CAF makers, αSMA, NG2 and laminin α3 chain, were significantly reduced in the α3KO-iCAFs as compared to iCAFs at both protein and transcriptional levels (Figure 5B,C). 

The α3KO-iCAFs were remarkably compromised in mechanical force exertion (Figure 5D). Embedded in collagen gels, α3KO-iCAFs contracted the gels by only 35 % as opposed to the contraction of 74 % by wild-type iCAFs (Figure 5E). The actomyosin system which is responsible for cell contraction is regulated inter alia by Rho proteins. The activity of RhoA was also reduced in α3KO-iCAFs (Figure 5F). This indicated that the integrin α3β1 did not necessarily affect the interaction of CAFs with collagen via collagen-binding integrins, but rather indirectly, by reinforcing the actomyosin machinery, such as αSMA production, and activation of actomyosin-orchestrating proteins, such as RhoA. These data demonstrated that integrin α3β1 is not only a maker of differentiation, but also is essential for sustained CAF differentiation by interacting with laminin-332. This interaction regulates the actomyosin machinery and cell contractility.

### 2.6. The Integrin α3β1-Mediated Interaction of CAFs with Laminin-332 Promote Invasion of Pancreatic Cancer Cells

**Figure 6:**
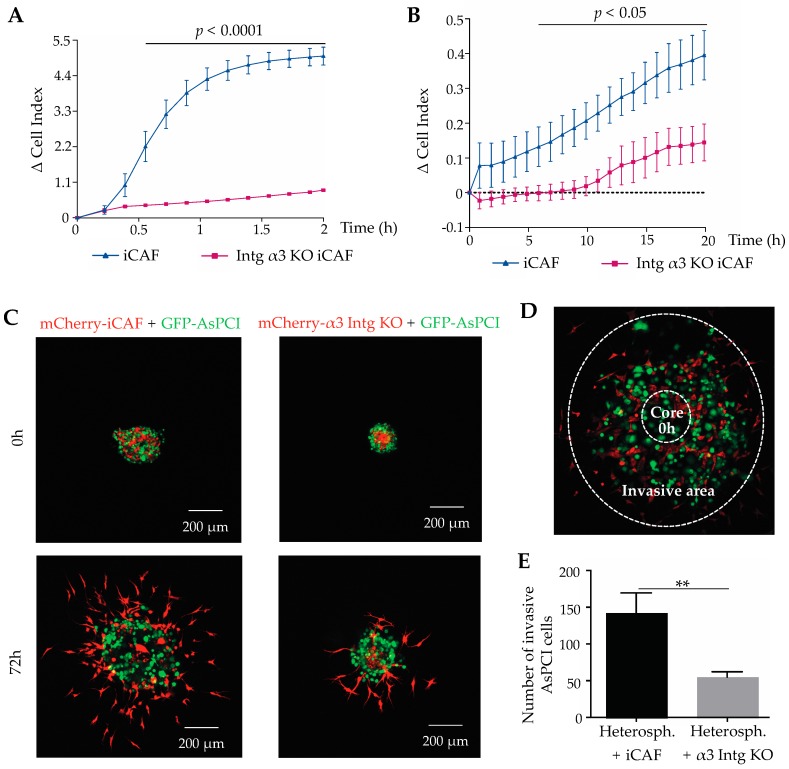
Regulated by the integrin α3β1 integrin-laminin-332 interaction, CAF differentiation determines adhesion to and haptotactic migration on laminin-332, as well as invasion not only of CAFs but also of carcinoma cells. (**A**) Adhesion to and (**B**) haptotactic migration on laminin-332 coated surfaces of α3KO-iCAFs were compared to these properties of iCAFs with impedance real-time measurements. After depletion of integrin α3β1, iCAFs scarcely interacted with immobilized laminin-332 and hence, almost abolished adhesion and migration. The time windows of significant differences in comparison to the iCAFs are shown as lines of the same colour above the plots, including the significance levels. Means ± SEM of three independent experiments were performed, and adhesion and migration values at every time point were compared by *t*-test. The time windows in which the adhesion signal differed significantly from the one of α3KO-iCAFs is shown above the plots with the significance levels denoted above the lines. (**C**) From heterospheroids, GFP-expressing AsPC-I cancer cells (green fluorescence) invaded into a surrounding gel containing collagen-I, matrigel components and laminin-332, within 72 h in significant higher numbers and along a larger distance, if they were combined with iCAFs (red) as compared to the integrin α3β1-deficient α3KO-iCAFs (red). (**D**) An invasive area was defined in the microscopic images as the ring area between an outer and inner perimeters given by the furthest invaded CAF and by the spheroid border at the beginning of the invasion experiments, respectively. (**E**) Quantification of invaded cancer cells from heterospheroids with iCAFs and integrin α3KO-iCAFs. Means ± SEM of three independent experiments are shown and compared by *t*-test (**, *p* < 0.01;).

The α3KO-iCAFs were tested for their adhesion and migration on laminin-332. They adhere significantly less to immobilized laminin-332 than wild-type iCAFs (Figure 6A). The haptotactic migration of α3KO-iCAFs through laminin-332-coated filters in the absence of any chemoattractant was significantly and persistently reduced over time (Figure 6B). These results rule out that other laminin-binding integrins or receptors can compensate for the lack of integrin α3β1 in cell adhesion to and migration along laminin-332-coated surfaces. To recapitulate the tumour bulk in an in vitro system, heterospheroids consisting of pancreatic duct adenocarcinoma AsPC-I cells and iCAFs or α3KO-iCAFs were embedded into gels which were composed of the ECM-components, type I-collagen, matrigel and laminin-332, typically found in tumour stroma. For distinction, the tumour cells had been transduced with GFP-encoding cDNA, whereas iCAFs and α3KO-iCAFs had been transduced with mCherry-encoding cDNAs (Figure 6C). Both iCAFs and AsPC-I cancer cells radially spread out from the heterospheroids and invaded the surrounding gel matrix. When the heterospheroids with the two different types of iCAFs were compared, α3β1 integrin-bearing iCAFs emigrated from the heterospheroids and invaded the gel more intensely than the α3KO-iCAFs. Cancer cells were considered to be invasive if they were detected within a circular area around the original core zone (Figure 6D). A significant larger number of AsPC-I cells invaded into the gel from the heterospheroid with wild-type iCAFs as compared to the heterospheroids with the α3KO-iCAFs (Figure 6E). In contrast, from homospheroids which are devoid of any iCAFs, AsPC-I cells do not invade the surrounding matrix (Figure S3), suggesting that iCAFs are a prerequisite for this cancer cell type to invade. Homospheroids of iCAFs invade the surrounding gel, however to a lesser extent than from heterospheroids, where they are combined with cancer cells, indicating that tumour cells might also increase the promigratory phenotype of iCAFs. The promigratory phenotype of iCAFs was additionally enhanced by the expression of integrin α3β1 (Figure S3). The heterospheroid invasion assay was also performed with another pancreatic duct epitheloid carcinoma cell line PANC-I, which was likewise labelled with GFP. The same effect was observed, that PANC-I cells invaded significantly more if co-cultured in heterospheroids with iCAFs, but not with α3KO-iCAFs (Figure S4). This corroborated the role of α3β1 integrin in the differentiation state of iCAFs, which correlates with α3β1 integrin expression, and the ability of iCAFs to promote the invasion of pancreatic cells. These experiments suggest that iCAFs and pancreatic cancer cells mutually promote their migration out the tumour spheroid and reinforce their invasion into the surrounding ECM. This cellular interplay depends on CAF differentiation, which in turn depends on α3β1 integrin expression in CAFs. Thus, α3β1 integrin and its interaction with ectopically expressed laminin-332 take a prominent role in supporting tumour cell invasion.

## 3. Discussion

Not only the tumour cells but also the tumour microenvironment, including the stromal ECM and the cancer-associated fibroblasts, play a crucial role in tumour progression. Our study sheds light on the key role of α3β1 integrin and its interaction with laminin-332 in the differentiation of CAFs, which consequently support tumour progression. Multiple factors influence the activation of fibroblasts, which develop a phenotype characterized by enhanced formation of actin stress fibres including αSMA. The latter is a marker for CAF differentiation, and it is an essential component of the force-generating actomyosin system of activated fibroblasts [32]. Our own observations and data published by others [33,34] have revealed that stiffness and tension of the adhesion substratum strongly influence cell behaviour and induce the transition from fibroblasts into myofibroblasts. [23,35] Moreover, release of TGF-β1 from the ECM depends on the tension that the cell exerts on their surrounding matrix [26,36]. To avoid these tension-dependent effects, we chose the spheroid culture model, in which cells form aggregates with each other without contacting the stiff plastic surface [37,38]. Furthermore, either individual cell types or a mixed population of different cell types can form homo- or heterospheroids respectively, recapitulating the bulk of solid tumours. Using homospheroids of iNFs and iCAFs, we show here, that CAF differentiation in this almost tension-free environment depends on TGF-β1. In addition, we demonstrate that CAF differentiation also depends on the interaction of integrin α3β1 with its ligand laminin-332. Lack of this interaction impaired TGF-β1-triggered iNF differentiation and reversed already differentiated CAFs into cells with normal fibroblast-like appearance. The CAFs used in this study were taken from tumour biopsies and immortalized with hTERT lentivirus as this immortalization protocol preserved genomic stability due to the maintenance of telomere integrity [39].

The carcinoma cell-free homospheroids of iCAFs contained all three chains of laminin-332, thereby resembling the presence of laminin-332 in the tumour stroma of pancreatic cancer in immunohistochemical sections. This is noteworthy in several aspects. Laminin-332 is generally considered to be of epithelial origin [7,40,41,42], where it is recruited into the anchoring filaments of the underlying basement membrane. In our study, it is ectopically found in the pancreatic tumour stroma. This is in line with few publications, where laminin-332 was preferentially localized not at the carcinoma cells, but in the neighbouring fibroblastic zone [28]. Moreover, iCAFs synthesize and deposit laminin-332 in the homospheroids, which lack any carcinoma cells, thereby demonstrating that CAFs can produce laminin-332. This has been shown for fibroblasts, which express laminin γ2 in a TGF-β-dependent manner [28]. As iCAFs produce TGF-β1 [43], this might be the autocrine stimulus for iCAFs in homospheroids to produce and secrete laminin-332. Another aspect is the detection of trimeric laminin-332 versus the detection of its individual monomeric chain. Our results demonstrate that CAFs synthesize all three laminin chains, α3, β3, and γ2. Many studies describe the secretion of trimeric laminin-332 by different carcinomas [5,7]. However, many publications also report the secretion of monomer chains of laminin-332, especially of the laminin β3 [31] and γ2 chain [40,44]. The latter is produced not only by epithelial and carcinoma cells [40,44,45], and its abundance in the blood of pancreatic cancer patients correlates with poor prognosis [30]. Our data show that CAFs produce all three laminin-332 chains which most probably assemble and are deposited as trimeric molecules within the tumour stroma, even in the absence of carcinoma in their vicinity and in the absence of exogenously added TGF-β1.

This study defines laminin-332 as a factor of the tumour microenvironment that, together with other soluble factors in the tumour microenvironment, sustains the phenotype of CAFs within the tumour stroma. In the onset of tumour progression, laminin-332 secreted by carcinoma cells, together with TGF-β1, triggers the transition of fibroblasts into CAFs. Upon differentiation, CAFs produce laminin-332, which, together with the laminin-332 produced by tumour cells, potentially contributes for the maintenance of their differentiation state. The up-regulation of laminin-332 can also be interpreted as a potential positive feedback loop, in which CAFs reinforces the differentiation of additional neighbouring NFs. The effective inhibition of CAF differentiation with the monoclonal antibody BM2 not only underlines the crucial role of laminin-332 in this process, but also points to the laminin-binding integrins as the essential receptors for CAF differentiation. We conclude this from the fact that BM2 only blocks the integrin binding site within the LG1-LG3 subdomains of laminin-332, but not any other potential interaction site, such as the heparan-sulfate proteoglycans binding site within the LG4-LG5 domain [46] or the putative binding site within the laminin γ2 chain for the collagen-binding α2β1 integrin [47]. In general, cellular interaction of laminin-332 is mediated predominately by integrins α3β1, α6β1, and α6β4. The monoclonal antibody GoH3, which antagonistically block the integrin α6 subunit [11], failed to reduce the interaction of laminin-332 with CAFs and did not alter CAF adhesion onto laminin-332, ruling out that an α6 subunit-containing integrin is involved in laminin-332-dependent CAF differentiation. Instead, our results highlight integrin α3β1 as the prime integrin receptor, which mechanistically enables CAFs to adhere to laminin-332 and cancer cell-deposited matrix. Moreover, integrin α3β1 triggers and sustains laminin-332-dependent CAF-differentiation. In line with this, we demonstrate that α3β1 integrin is significantly upregulated during CAF differentiation and therefore is a potential CAF marker. The finding, that CAFs lose their phenotype upon knock-out of the integrin α3 subunit, proved the α3β1 integrin-dependent mechanism. Moreover, it highlights that α3β1 integrin is not only a marker for the CAF phenotype but also a key player in laminin-332-dependent CAF differentiation.

The integrin α3 gene knock-out experiments revealed an altered CAF phenotype, with diminished level of αSMA within stress fibres and diminished activity of RhoA, which are both in line with the observed decrease of contractility of the integrin α3β1-deficient iCAFs. As integrin α3β1 does not bind to collagen-I [12], the collagen-binding α2β1 integrin is likely responsible of force transmission to the collagen fibrils and hence mediate contraction of collagen gels by iCAFs. However, the amount of α2β1 integrin did not change when the integrin α3β1 was knocked-out. These findings suggest that the force-generating actomyosin system, but not the force transmitting collagen-binding integrins, was responsible of the gel contraction. The α3KO-iCAFs showed similar characteristics to the iNFs phenotype with respect to CAF differentiation markers and laminin-332 production, underlining the essential role of α3β1 integrin in inducing and maintaining the differentiated CAF state.

Recapitulating the tumour bulk in vitro, the heterospheroids of pancreatic duct adenoma carcinoma cells and iCAFs demonstrate that the carcinoma cells and neighbouring CAFs influence each other in a mutually interdependent manner, such as CAFs stimulate invasion of carcinoma cells [20]. Also during invasion, integrin α3β1 of CAFs plays a crucial role, as its presence in CAFs increased the invasion radius and number of invading carcinoma cells in gels mimicking the tumour ECM. Integrin α3β1 deficiency diminishes tumour cell invasion most likely by reversing the CAF phenotype and by reducing the activity of the actomyosin system. It has been previously observed that integrin α3β1 is required in fibroblasts for squamous carcinoma cell invasion [48]. Although the latter is a collective cancer cell invasion, integrin α3β1 of CAFs takes a similarly important role in single cell invasion of pancreatic carcinoma as we demonstrate in this work.

## 4. Materials and Methods

### 4.1. Cell Lines and Pancreatic Adenocarcinoma Sections

Freshly isolated human pancreatic CAFs from resected pancreatic ductal adenocarcinoma, Union for International Cancer Control (UICC) Stage 3, were prepared as previously described [49]. Briefly, fresh stromal specimens were dissected from pancreatectomies and washed with Hank’s Balanced Salt Solution (HBSS, Lonza, Basel, Switzerland). The tissue was minced and digested with 1 mg/mL collagenase II (Worthington Bichemicals, Lakewood, NJ, USA) at 37 °C under constant shaking followed by filtration through a 70 μm nylon mesh (Sigma-Aldrich, St. Louis, MO, USA). The tumour tissue-derived CAFs and the commercially obtained normal pancreatic fibroblasts (PELOBiotech, Martinsried, Germany) were cultivated in MEM (Sigma-Aldrich) containing 10% heat-inactivated fetal bovine serum (FBS, Thermo Fisher Scientific, Waltham, MA, USA) and 1% penicillin/streptomycin (Thermo Fisher Scientific). The AsPC-I (ATCC, Manassas, VA, USA) were cultivated in RPMI1640 (Lonza) containing 10% FBS, 1% penicillin/streptomycin and 1% sodium pyruvate (PAA, The Cell Culture Company, Cambridge, UK). The PANC-I (ATCC) and the HEK293 were cultivated in DMEM containing 10% FBS, 1% penicillin/streptomycin. Human tissue was handled in accordance to principles outlined in the Declaration of Helsinki and approved by the ethics committee of the University of Muenster (approval nr. 2016-074-f-S).

### 4.2. Immortalization of Human Pancreatic Fibroblasts and CAFs

Primary CAFs and normal pancreatic fibroblasts were immortalized through the expression of hTERT. The hTERT-encoding vector was transduced into these target cells with commercially available high titre lentivirus (Cellomics Technology, Halethorpe, MD, USA). The target cells at a density of 75–100 × 10^3^ per well were transduced with serum and antibiotics free MEM containing virus particles at a multiplicity of infection of 10. After incubation with the viral solution for 1 h, at 37 °C and 5% CO_2_, MEM containing 10% FBS and 1% penicillin/streptomycin was added to the cells. After 24 h, the MEM supernatant was exchanged. After 72 h transduced cells were selected in medium containing of geneticin (Thermo Fisher Scientific), initially 75 µg/mL, then raising to 150 µg/mL over the following weeks.

### 4.3. Immunostaining of Human Pancreatic Adenocarcinoma Sections

Sections of human pancreatic adenocarcinoma from biopsies taken from patients during surgery in consent with the local ethic committee (approval nr. 2015-102-f-S) were fixed with PFA (Honeywell Speciality Chemicals Seelze, Seelze, Germany) for 30 min at room temperature (RT), and permeabilized with Triton-X (Sigma-Aldrich) for 4 min at RT. The samples were blocked with 10% FBS and 2% BSA (AppliChem, Darmstadt, Germany) in PBS for 2 h at RT. The primary antibodies directed against laminin-332 α3 subunit, tenascin-W, laminin-332 (Abcam, Cambridge, UK) (20 µg/mL) or NG2 (10 µg/mL), were incubated for 90 min at RT in blocking buffer. The specimens were incubated with the secondary antibody (all purchased from Thermo Fisher Scientific): Alexa-488 and -568 anti-mouse, Alexa-488 and -568 anti-rabbit (10 µg/mL) for 90 min at RT in blocking buffer. The cell nuclei were staining with DAPI for 4 min at RT. The sections were preserved with mounting medium (Agilent, Santa Clara, CA, USA). Images were acquired using the confocal microscope (LSM 700 and LSM 800, Zeiss, Oberkochen, Germany).

### 4.4. 3D Spheroids as In Vitro Model for Fibroblast Differentiation and Their Immunofluorimetric Analysis

Spheroids were formed from 750–1000 freshly thawed cells, human iNFs of passage 9–12, iCAFs of passage 15–24 or integrin α3 KO iCAFs 22–27, in a non-adherent round bottom 96-well plate method. Cells were resuspended in a solution composed of ¼ volume of a 3 g/mL methylcellulose (Sigma-Aldrich) solution and ¾ volume of cell culture media and respective treatments. 100 µL of this suspension was distributed per well. The iNFs were treated with 10 ng/mL TGF-β1 (PeproTech, Hamburg, Germany), 10 ng/mL TGF-β1 + 20 µg/mL BM2 or 10 ng/mL TGF-β1 + 10 µg/mL lebein-1. The iCAFs were treated with 20 μg/mL BM2, 10 μg/mL lebein-1 or 20 μg/mL BM2 + 10 μg/mL lebein-1. To immunofluorimetrically detect αSMA and NG2 at least 3 replicates were stained, for each condition. All staining steps were performed in 1.5 mL reaction tubes with centrifugation steps in between. The spheroids were fixed with methanol for 10 min at −20 °C for laminin-332 and tenascin-W, or with 4% PFA for 20 min at RT for αSMA and NG2. They were permeabilised with 0.1% of Triton X-100 for 4 min at RT and incubated with blocking buffer (PBS containing 5% FBS and 2% BSA) for 1h at RT. The primary antibodies were added in staining buffer (PBS, 2.5% FBS and 1% BSA) at 4 °C overnight, followed by the secondary antibody in staining buffer at RT for 1h 30 min. The following primary antibodies used were: anti-αSMA Cy-3 conjugated (Sigma-Aldrich), anti-NG2 (Millipore, Burlington, MA, USA) (5 µg/mL); BM2 anti-laminin α3, 6F12 anti-laminin α3 (20 µg/mL). The following secondary antibodies were used: Alexa 488 anti-rabbit and Alexa 488 anti-mouse (5 µg/mL). The cells were incubated with DAPI staining solution for 4 min at RT. Between each of these steps, spheroids were washed with PBS three times. Images were acquired using the confocal microscope (LSM 700, Zeiss).

### 4.5. Quantification of Gene Expression in Homospheroids by RT q-PCR

Approximately 300 spheroids were collected, washed with PBS and dissociated with collagenase B solution (4–8 mg/mL Collagenase B, 50 μg/mL DNase I, 2% BSA, 1 mM CaCl_2_) at 37 °C for about 30–45 min. Total RNA was isolated with the RNeasy Mini Kit (Qiagen, Hilden, Germany) and cDNA was produced with the QuantiTect Reverse Transcription Kit (Qiagen) according to manufacturers’ instructions. Transcription levels were quantified in duplicate by real-time PCR (Rotor-Gene Q) with QuantiFast SYBR Green PCR Kit (Qiagen). Ct values were corrected for the Ct-value of the endogenous reference gene (TOP-1). ΔΔCt was determined as: ΔΔCt = average ΔCt (sample of interest) − average ΔCt (reference control). The primers used were: α-SMA: Fw 5′-CCGACCGAATGCAGAAG GA-3′, Rev 5′-ACAGAGTATTTGCGCTCCGAA-3′ [50]; Laminin α3 chain: Fw 5′-GCTCAGCTGTTTGTGGTTGA-3′, Rev 5′-TGTCTGCATCTGCCAATAGC-3′ [51]; Laminin β3 chain: Fw 5′-GGCAGATGATTAGGGCAGCCGAGGAA-3′ Rev 5′-CGGACCTGCTGGA TTAGGAGCCGTGT-3′ [31]; Laminin γ2 chain: Fw 5′-GATGGCATTCACTGCGAGAAG-3′ Rev 5′-TCGAGCACTAAGAGAACCTTTGG-3′ [51]; NG2: Fw 5′-CTGCAGGTCTATGTCGGTCA-3′ Rev 5′-TTGGCTTTGACCCTGACTATG-3′ [52]; TOP-1: Fw 5′-CCAGACGGAAGCTCGGAAAC-3′ Rev 5′-GTCCAGGAGGCTCTATCTTGAA-3′ [53].

### 4.6. Collagen I Gel Contraction Assay

The collagen I gel was prepared by adding 15 mM solution of NaOH to 1 mg/mL of rat tail collagen I (Thermo Fisher Scientific), diluted in MEM medium containing 10% FBS. 180 × 10^3^ freshly thawed iNF, iCAFs and integrin α3 KO iCAFs were mixed with the collagen I solution. If applied, BM2 or lebein-1 were added to the collagen I containing-CAFs suspension at 20 µg/mL and 10 µg/mL, respectively. After 30 min of incubation at RT, the gel was detached from the well wall with a scalpel. MEM medium containing 10% FBS, 1% penicillin/streptomycin was added to the gel.

### 4.7. Flow Cytometry Analysis of Integrin Expression in Human Pancreatic Fibroblasts and CAFs

Spheroids were dissociated with collagenase, as described in Section 4.5, incubated in blocking buffer containing 2% horse serum and 0.1% BSA for 20 min at RT and, subsequently stained for 30 min with primary integrin subunit-directed antibodies and corresponding secondary antibodies, each diluted at 10 μg/mL in the blocking buffer with intermittent and final three with PBS + 0.1% BSA. The stained cells were analysed in the BD FACSCalibur. 

### 4.8. Real-Time Adhesion and Migration of Fibroblasts and CAFs Using xCELLigence

Adhesion and migration of iNFs, iCAFs and integrin α3 KO iCAFs were recorded by impedance measurement with E- and CIM-plates, respectively, in the xCELLigence system (ACEA Biosciences Inc., San Diego, CA, USA). The impedance values are given in the arbitrary unit of cell index (CI) and depend on electrode coverage by cells [54]. For monitoring adhesion, E-plates were coated with 5 µg/mL of laminin-332 (Biolamina, Sundbyberg, Sweden) overnight at 4 °C and blocked with 0.1% BSA for 1 h at RT. To produce an AsPC-I cell-deposited ECM, AsPC-I cells were cultivated in the E-plate wells for 3–4 days, and then solubilized with 1% (*v*/*v*) Triton X100-solution in PBS containing 1 μg/mL aprotinin/leupeptin (Carl Roth, Karlsruhe, Germany) and 2 mM PMSF (Carl Roth)] for 1 h at 37 °C. The wells were washed once and incubated for 1 h at 37 °C with a solution of 2 M Urea and 1 M NaCl. The cells were seeded at a density of 1 × 10^5^ per well. Different inhibitors were used: BM2, 20 µg/mL, lebein-1, A3IIF5 or GoH3 (BD Biosciences, Franklin Lakes, NJ, USA), 10 µg/mL each. To monitor haptotactic migration, CIM-plates were coated with 5 µg/mL laminin-332 overnight at 4°C. The next day, 1 × 10^5^ CAFs or integrin α3 KO iCAFs were seeded, in serum-free media. Both adhesion and migration experiments were performed at 37 °C with 5% CO_2_. Subtraction of the corresponding background value yielded Δ cell index values for all time points. Data were evaluated with the RTCA software 1.2.

### 4.9. Knocking out the Integrin α3 Subunit in Human Pancreatic CAFs

The gene encoding the α3 subunit was knocked-out in iCAFs, by using the CRISPR-Cas9 technology [55]. Two sgRNAs, designed and selected with the CRISPR design website (http://crispr.mit.edu/), were employed to increase the chance of deleting the targeted DNA sequence. The human integrin α3 subunit-targeting sgRNA were: Sg1 top 5′CACC GCTACTCGGTCGCCCTCCAT3′ bottom 5′AAAC ATGGAGGGCGACCGAGTAGC3′ and Sg2 top 5’CACC GCTCTGTGCGCTCGCCTTGA3′ bottom 5′AAAC ATGGAGGGCGACCGAGTAGC3′. The oligos were cloned into a lentiCRISPRv2 plasmid (Addgene, 52961, Cambridge, MA, USA) and amplified in *E. coli* strain Stbl3. The vector was isolated with the kit NucleoBond® Xtra Maxi (Macherey&Nagel, Düren, Germany) and validated by sequencing. To generate the lentivirus, 6 × 10^5^ HEK293 cells were plated in high glucose DMEM, 10% FBS, without penicilin/streptromycin. After 24 h, 40 µg/mL of PEI, 4.8 µg of sgRNA-encoding lentiCRISPRv2 vector, 3.6 µg of each psPAX2 and pCMV-VSV-G plasmid were mixed in Opti-MEM (Thermo Fisher Scientific), added to the HEK293 cells and incubated at 37 °C for 24 h. Thereafter, transfection media was replaced with high glucose DMEM containing 10% FBS and 1% penicillin/streptromycin. The lentivirus-containing supernatant was filtrated through a 0.45 µm filter and diluted in MEM supplement free media in a ratio of 1:1 to transduce iCAFs. Cells were simultaneously transduced with the two different virus-containing CRISPR vectors at a 1:1 ratio. The subsequent steps are outlined in Section 4.2. The integrin α3β1 deficient cell subpopulation was enriched by flow cytometric cell sorting.

### 4.10. Determination of RhoA Activation in CAFs and Integrin α3 KO CAFs

RhoA activation was analysed with a RhoA pull-down Activation assay kit (Cytoskeleton Inc., Denver, CO, USA) according to the manufacturer’s instructions. AsPC-I-deposited ECM was used to stimulate RhoA activation. iCAFs and α3 KO iCAFs were plated onto the ECM, at a density of 2 × 10^6^ cells per 10 cm dish and collected 30 min later. Beads functionalized with the rhotekin domain pulled down activated RhoA from the cell lysate. RhoA in the eluate from these beads was electrophoretically separated in a 12% SDS-PAGE gel, immunoblotted and detected with Pierce™ ECL Western Blotting Substrate (Thermo Fisher Scientific) in the Image Quant LAS 4000 (GE Healthcare, Freiburg, Germany).

### 4.11. Invasion Assay

The interplay of CAFs and AsPC-I during infiltration of the surrounding matrix was studied with heterospheroids embedded in 3D gels made of ECM components. The iCAF and α3KO-iCAFs were transduced with lentivirus containing a mCherry vector, and AsPC-I and PANC-I cells with a lentivirus containing a GFP vector. Both vectors were kindly provided by Dr. Stephan Huveneers. The transduction was performed as detailed in Section 4.2, and the virus was produced as described in Section 4.9 and concentrated using the kit Lenti-X™ Concentrator (Takara Bio Inc., Shiga, Japan). The transfected iCAFs, AsPC-I and PANC-I cells were selected with puromycin (see Section 4.9). Being already resistant to puromycin, α3KO-iCAFs were sorted by fluorescence-assisted cell sorting. The heterospheroids comprised 400 mCherry-iCAFs or mCherry-α3KO-iCAFs as well as 400 GFP-AsPC-I or GFP-PANC-I cells. The homospheroids also comprised 400 of each cell type. The spheroids were embedded into a gel composed of 2 mg/mL of rat tail collagen I, 30% Matrigel (Thermo Fisher Scientific) and 2.5 µg/mL of laminin-332, on a µ-Slide angiogenesis chamber (Ibidi, Martinsried, Germany). The gel was overlaid with cell culture medium and images were acquired using the confocal microscope (LSM 800, Zeiss). The experiment was analysed for the number of invading AsPC-I cancer cells (Figure 6D).

### 4.12. Immunofluorescence Quantification

The immunofluorescence images were quantitively analysed with ImageJ software as published previously [56]. As background, the integrated signal density of a cell-free region of interest (ROI) was determined. The measured integrated signal density is the sum of the intensity values of the pixels within the selected ROI. The total corrected cell fluorescence (TCCF) was calculated as: TCCF = integrated signal density − (area of selected cell × mean fluorescence of background). The total corrected fluorescence (TCF) of the spheroids was determined as the TCCF normalized to the area of spheroid and the number of stacks. This accounted for their varying sizes and variance in spherical shape.

### 4.13. Statistics

Statistical analyses were performed with the GraphPad Prism 6 Software (GraphPad Software, San Diego, CA, USA). Comparisons between two groups were conducted using the Student’s *t* test. Unless stated otherwise, *p* values smaller than 0.05 were considered significant. The data was presented as mean ± standard error of the mean (SEM).

## 5. Conclusions

This study highlights integrin α3β1 and the interaction with its ligand laminin-332 as a key event in CAF differentiation and in the interplay of carcinoma cells with fibroblast in the tumour stroma. It can be envisioned that pancreatic duct cells by secreting laminin-332 induce TGF-β1-stimulated fibroblasts in their vicinity to differentiate into CAFs. Thus, they selectively upregulate integrin α3β1 among the laminin-binding integrins and consequently make themselves more responsive towards laminin-332. Noteworthy, we found strong ectopic production of laminin-332 at invasive fronts of the tumour, where the cancer cells border on normal tissue. Laminin-332 is relevant for CAFs to sustain their phenotype. These CAFs help the carcinoma cells to invade the surrounding ECM, supporting spreading and metastasis of the pancreatic duct adenocarcinoma, which is commonly observed in pancreatic cancer and the main cause for its lethality.

## Supplementary Materials

The following are available online at http://www.mdpi.com/2072-6694/11/1/14/s1, Figure S1: Double treatment of iCAFs with both BM2 and lebein1 did not yield a synergistic effect as compared to single treatment with either BM2 or lebein-1, Figure S2: Flow cytometric expression analysis of integrin α3, α6, α2 and β1 subunits to compare iCAFs and integrin α3KO-iCAFs, Figure S3: Progression of control homospheroids consisting of only one cell type (mCherry-iCAFs, mCherry-α3KO-iCAFs and GFP-AsPC-I) in the spheroid invasion assay, for comparison to heterospheroids (Figure 6C), Figure S4: The invasion of GFP-labelled PANC-I also depended on neighbouring mCherry-labelled iCAFs in an α3β1 integrin-dependent manner in the spheroid invasion assay, similarly to the invasion of AsPC-I cells.

## Abbreviations

αSMA	α-Smooth Muscle Actin
CAF	Cancer-Associated Fibroblast
ECM	Extracellular Matrix
FBS	Fetal Bovine Serum
hTERT	Human Telomerase Reverse Transcriptase protein
iNF	Immortalized Normal Fibroblast
iCAF	Immortalized Cancer-Associated Fibroblast
α3KO-iCAF	Integrin α3 subunit Knock-Out immortalized Cancer-Associated Fibroblasts
Leb1	Lebein-1
MEM	Minimal Essential Medium
NG2	Neural/Glial antigen 2
RT	Room Temperature
TCF	Total Corrected Fluorescence
TCCF	Total Corrected Cell Fluorescence
TGF-β1	Transforming Growth Factor-β1
