# Supplementary material for: The Interaction between Laminin-332 and α3β1 Integrin Determines Differentiation and Maintenance of CAFs, and Supports Invasion of Pancreatic Duct Adenocarcinoma Cells
Source: Cancers (Basel). 2018 Dec 21;11(1):14. doi: 10.3390/cancers11010014 (PMC6356648; doi:10.3390/cancers11010014)
Supplement: Supplementary file 1 [file cancers-11-00014-s001.pdf]

# Supplementary Materials: The Interaction Between Laminin-332 and α3β1 Integrin Determines Differentiation and Maintenance of CAFs, and Supports Invasion of Pancreatic Duct Adenocarcinoma Cells

**Ana C. Martins Cavaco, Maryam Rezaei, Michele F. Caliandro, Augusto Martins Lima, Martin Stehling, Sameer A. Dhayat, Jörg Haier, Cord Brakebusch, Johannes A. Eble**

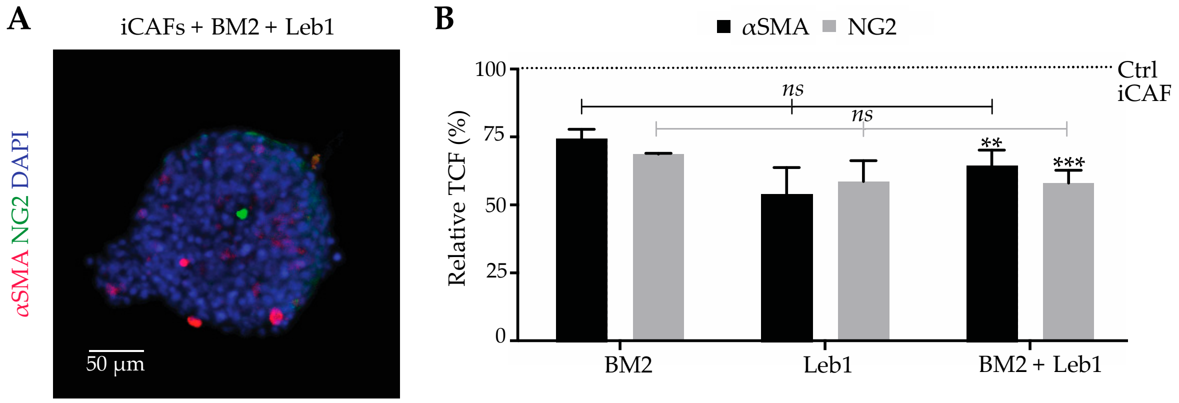


**Figure S1.** Double treatment of iCAFs with both BM2 and lebein1 did not yield a synergistic effect as compared to single treatment with either BM2 or lebein-1. (**A**) As in Figure 4A, spheroids of iCAFs were treated with both BM2 (20 µg/mL) and lebein1 (10 µg/mL) for 12 h. (**B**) After immunofluorimetric staining, αSMA and NG2 were quantified and normalization to the corresponding signals of non-treated iCAF spheroids. Means ± SEM of three independent experiments are shown and compared with non-treated iCAF spheroids with a *t*-test (**, $p < 0.01$; ***, $p < 0.001$) Both CAF-markers were significantly reduced. However, the reduction of the double treatment with BM2 and lebein-1 did not significantly differ from the signal reductions observed for the individual treatment with either of the inhibitor of integrin-laminin-332 interaction. The values of individual treatment do not differ from the values of double treatment, ruling out that cellular interaction with laminin-332 via receptors other than laminin-binding integrins play a major role.

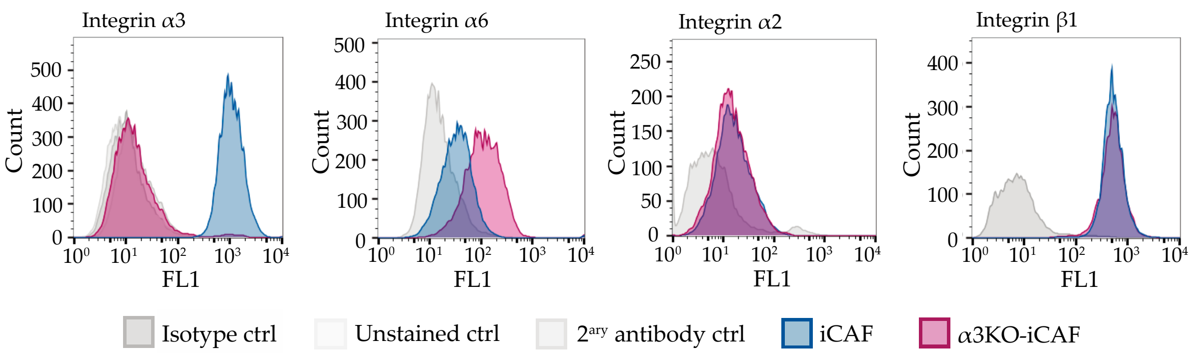


**Figure S2.** Flow cytometric expression analysis of integrin α3, α6, α2 and β1 subunits to compare iCAFs and integrin α3KO-iCAFs. After fluorescence-assisted cell sorting, a pure population of integrin α3β1 knocked-out iCAFs was obtained. This histogram presents three different controls, to assure that the population is indeed knock-out: isotype control, unstained cells control and secondary antibody control. The α3KO-iCAFs appear to up-regulate α6 integrin slightly. Integrin α2 subunit expression was not altered by the knock-out of the integrin α3 subunit, neither was the β1 expression. These three experiments were performed with the isotype control.

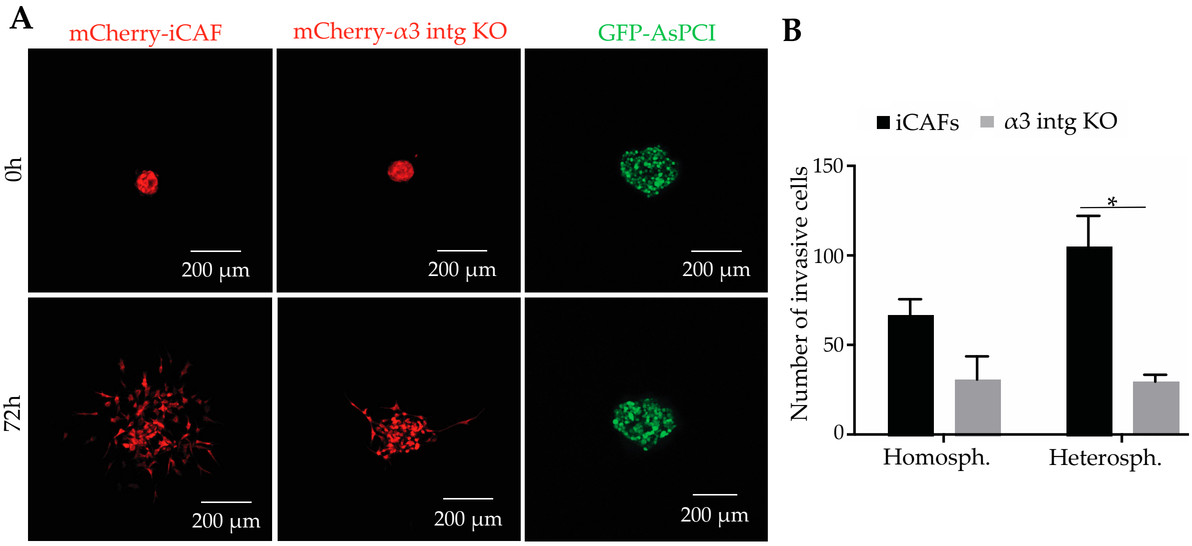


**Figure S3.** Progression of control homospheroids consisting of only one cell type (mCherry-iCAFs, mCherry-α3KO-iCAFs and GFP-AsPC-I) in the spheroid invasion assay, for comparison to heterospheroids (Figure 6C). (**A**) mCherry-iCAFs (red) were able to invade the surrounding gel. So are mCherry-α3KO-iCAFs (red), although in lower numbers. In contrast, GFP-AsPC-I cells (green) failed to leave the spheroid, unless when co-cultured with mCherry-iCAFs or with mCherry-α3KO-iCAFs (Figure 6C). The latter stimulated less GFP-AsPC-I cells to invade the gel as shown in figure Figure 6E. (**B**) The number of invasive mCherry iCAFs and mCherry-α3KO-iCAFs were compared when in homospheroids or in heterospheroids. The invasive cells were count as shown in Figure 6D. Means ± SEM of three independent experiments are shown and compared by t-test (*, $p < 0.05$).

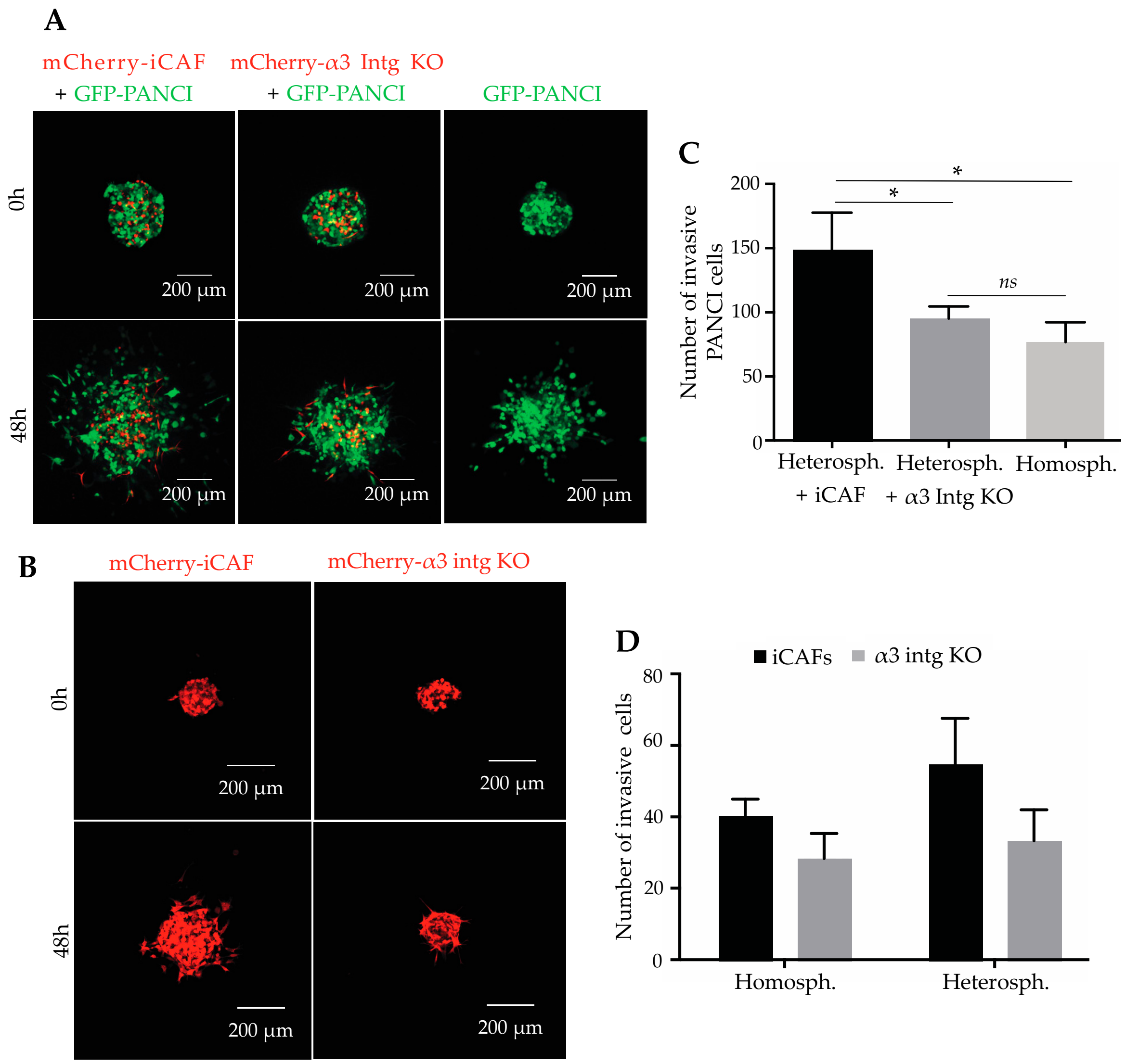


**Figure S4.** The invasion of GFP-labelled PANC-I also depended on neighbouring mCherry-labelled iCAFs in an α3β1 integrin-dependent manner in the spheroid invasion assay, similarly to the invasion of AsPC-I cells. (**A**) Although the GFP-PANC-I cells invade the surrounding gel from homospheroids, this invasion was further increased when the cancer cells were co-cultures in heterospheroids with the mCherry-iCAF. In contrast, mCherry-α3KO-iCAFs failed to support PANC-I cells invasion significantly. Invasion was measured after 48h due to the higher invasiveness of PANC-I cells. (**B**) The invasion of control homospheroids made of mCherry-iCAFs and mCherry-α3KO-iCAFs, alone. (**C**) Biometric evaluation of invading PANC-I cancer cells. As compared to the homospheroids of PANC-I, the number of invaded cancer cells differed significantly, only if they were cultured in heterospheroids with wild- type iCAFs, but not with integrin α3β1-deficient iCAFs. (**D**) The numbers of invasive mCherry iCAFs and mCherry-α3KO-iCAFs were compared from homospheroids and heterospheroids. Although remarkably more iCAFs, especially the integrin α3β1-bearing iCAFs, invaded from heterospheroids than from homospheroids, suggesting also a promigratory influence of cancer cells on iCAFs, these differences did not reach significance level. The invasive cells in images similar to the ones in (**A**) and (**B**) were count as shown in Figure 6D. Means ± SEM of three independent experiments are shown in (**C**) and (**D**), and compared by t-test (*, $p < 0.05$).
